# Hypodermic Quinia

**Published:** 1880-07

**Authors:** 


Hypodermic Quinia.—A Russian doctor, in St. Petersburg Medical Gazette, recommends the hypodermic injection of quinine in the treatment of certain fevers in which the alkaloid is indicated, on account of the certainty and rapidity of its action. Besides, quinine thus administered had upon the uterine contractions in confinements a powerful and promp influence. M. Smoliskii employs from preference chlorohydrate of quinine which did not crystallize at a moderate temperature. He dissolves it in distilled water.

